# Deficiency of cold‐inducible RNA‐binding protein exacerbated monocrotaline‐induced pulmonary artery hypertension through Caveolin1 and CAVIN1

**DOI:** 10.1111/jcmm.16437

**Published:** 2021-03-23

**Authors:** Jingjing Liu, Xianting Ke, Luxin Wang, Yangyang Zhang, Jian Yang

**Affiliations:** ^1^ Key Laboratory of Arrhythmias, Ministry of Education Shanghai East Hospital Tongji University School of Medicine Shanghai China; ^2^ Department of Cardiology Shanghai East Hospital Tongji University School of Medicine Shanghai China; ^3^ Institute of Medical Genetics Tongji University School of Medicine Shanghai China; ^4^ Department of Cardiovascular Surgery Shanghai East Hospital Tongji University School of Medicine Shanghai China

**Keywords:** Caveolin1, CAVIN1, cold‐inducible RNA‐binding protein, monocrotaline, pulmonary artery endothelial cells, pulmonary artery hypertension

## Abstract

Cold‐inducible RNA‐binding protein (CIRP) was a crucial regulator in multiple diseases. However, its role in pulmonary artery hypertension (PAH) is still unknown. Here, we first established monocrotaline (MCT)‐induced rat PAH model and discovered that CIRP was down‐regulated predominantly in the endothelium of pulmonary artery after MCT injection. We then generated *Cirp*‐knockout (*Cirp*‐KO) rats, which manifested severer PAH with exacerbated endothelium damage in response to MCT. Subsequently, *Caveolin1* (*Cav1*) and *Cavin1* were identified as downstream targets of CIRP in MCT‐induced PAH, and the decreased expression of these two genes aggravated the injury and apoptosis of pulmonary artery endothelium. Moreover, CIRP deficiency intensified monocrotaline pyrrole (MCTP)‐induced rat pulmonary artery endothelial cells (rPAECs) injuries both in vivo and in vitro, which was counteracted by *Cav1* or *Cavin1* overexpression. In addition, CIRP regulated the proliferative effect of conditioned media from MCTP‐treated rPAECs on rat pulmonary artery smooth muscle cells, which partially explained the exceedingly thickened pulmonary artery intimal media in *Cirp*‐KO rats after MCT treatment. These results demonstrated that CIRP acts as a critical protective factor in MCT‐induced rat PAH by directly regulating CAV1 and CAVIN1 expression, which may facilitate the development of new therapeutic targets for the intervention of PAH.

## 1 INTRODUCTION

Pulmonary artery hypertension (PAH) is a cardiovascular disease with high morbidity and mortality,^1^ characterized by increased pulmonary artery pressure and vascular resistance.^2^, ^3^ The initial pulmonary endothelial cells injury, subsequent smooth muscle cells proliferation and eventually irreversible vascular remodelling and resistance are widely recognized as main pathogenesis of the disease.^4^, ^5^ However, because of insufficient knowledge about the aetiology and pathogenesis of PAH, the development of new strategies in diagnosis and treatment of the disease is severally restricted.^6^

Cold‐inducible RNA‐binding protein (CIRP) is a widely expressed RNA‐binding protein, involved in various important cellular processes such as gene expression, genomic stability, apoptosis and proliferation.^7^ So far, stimuli such as cold stress and short‐term exposure to hypoxia have been found to increase CIRP expression, whereas heat stress and long‐term exposure to hypoxia down‐regulate its expression.^8^ Therefore, it has been reported that CIRP plays important roles in various diseases.^7^ However, the effect of CIRP on PAH has not been investigated.

To determine the expression and function of CIRP in monocrotaline (MCT)‐induced PAH and to investigate its downstream regulatory molecules and the related mechanisms in this process, we established MCT‐induced rat PAH model, in which endothelium damage was the main cause of the disease.^9^, ^10^ CIRP was down‐regulated in endothelium of MCT‐induced PAH, and severer disease phenotype manifested in *Cirp*‐knockout (KO) rats. Moreover, *Caveolin1* (*Cav1*) and *Cavin1* were validated as downstream targets of CIRP, the decreased expression of these two genes aggravated apoptosis and injury of pulmonary artery endothelium. These data suggested that CIRP is critical for the homeostasis of endothelium and may serve as new therapeutic target for PAH.

## 2 METHODS

### 2.1 Animals

*Cirp*‐KO rats were generated in sprague dawley (SD) background, which has been described in detail previously,^11^ and the deletion of *Cirp* was verified by both quantitative PCR (qPCR) and Western blotting. All procedures conducted in rats were in accordance with the NIH Guidelines for Care and Use of Animals in Research (NIH Publication NO. 85‐23, revised in 1996) and were approved by the Institutional Animal Care and Use Committee at Tongji University School of Medicine.

Male *Cirp*‐KO and wild‐type (WT) rats weight 180‐250 g were fed with normal foods and maintained under specific pathogen‐free conditions. Rats received a single subperitoneal injection of MCT (60 mg/kg; Sigma) to induce PAH, whereas the control rats were injected with a single dose of saline solution (0.9% NaCl). The animals were subjected to transthoracic echocardiography under anaesthesia and blood pressure measurements in consciousness, both at week two and four after MCT injection.

Pulsewave doppler echocardiology was performed to record the pulmonary blood outflow at the level of the aortic valve in the short‐axis view to measure pulmonary acceleration time (PAT) and pulmonary ejection time (PET) using a Visual Sonics Vevo 770 ultrasound (Visual Sonics) equipped with a 20‐MHz linear array transducer. Transthoracic M‐mode and two‐dimensional echocardiography using a Visual Sonics Vevo 770 ultrasound equipped with a 30‐MHz linear array transducer were performed at parasternal long‐axis view to record left ventricular anterior and posterior wall thicknesses and left ventricular internal diameters at end‐systole and end‐diastole, which were used to calculate fractional shortening (FS) and the ejection fraction (EF) of left ventricle according to standard formulas.

The systole and diastole blood pressure of rats in quiet environments without outside stimulations was measured noninvasively with coda monitor, and mean artery pressure (MAP) was calculated according to the following formula: MAP = 1/3 systole pressure + 2/3 diastole pressure.

At week four after MCT injection, the animals were anaesthetized, and right ventricular systolic pressure (RVSP) was measured by inserting a pressure‐volume catheter into the right ventricle. Subsequently, rats were sacrificed; after effectively cleaning the remaining blood with heparin saline, the lungs were harvested, snap‐frozen in liquid nitrogen, and then stored at −80°C for RNA and protein extraction. To assess the right ventricular hypertrophy, right ventricle (RV) and left ventricle plus septum (LV + S) were dissected and weighed, respectively.

### 2.2 Immunohistochemistry

The dissected lungs were fixed in PBS/4% paraformaldehyde (PFA) (Sigma) for 48 hours, dehydrated with graded ethanol (30%, 50%, 75%, 95%, 100%) and embedded in paraffin. Sections (4 µm) were blocked with PBS/6% goat serum (Gibco) at room temperature for 45 minutes, incubated with anti‐CIRP antibody (Proteintech), anti‐CD31 antibody (Abcam), anti‐CD68 antibody (Santa Cruz), anti‐SMA antibody (Genetex), anti‐CAV1 antibody (Abcam) or anti‐CAVIN1 antibody (Abcam) at 4°C overnight and washed with PBS/0.1% Tween (PBST). Subsequently, for diaminobenzidine (DAB) staining, the sections were incubated with horseradish peroxidase (HRP)‐conjugated goat anti‐rabbit IgG (Beyotime) at room temperature for 30 minutes and then treated with DAB peroxidase substrate solution (Beyotime) according to the manufacture's instruction for colour development. For Alexa Fluor staining, the sections were incubated with Alexa Fluor 488 conjugated goat anti‐rabbit IgG (Abcam) or Alexa Fluor 555 conjugated goat anti‐mouse IgG (Abcam) at room temperature for 1 hour. Nuclei were stained with DAPI (Sigma). The images were captured with fluorescence microscope (Leica).

### 2.3 Cell culture and transfection

Rat pulmonary artery endothelial cells (rPAECs) and rat pulmonary artery smooth muscle cells (rPASMCs) purchased from Creative Bioarray Co. were cultured in DMEM with 5% FBS (Excell) and used for experiments between passage three and nine.

For gene knockdown, cells were transfected with siRNAs against *Cirp*, *Cav1* and *Cavin1* by Lipofectamine RNAiMAX (Invitrogen). Scramble siRNA was used as negative control (si‐*NC*). The siRNA sequences used are listed in Table 1.

**TABLE 1:** siRNA sequences used in this article

Construct	Sequence (5′‐3′)
si*‐NC*	UUCUCCGAACGUGUCACGUTT
si*‐Cirp*	GGGUUUGUCACCUUUGAAATT
si*‐Cav1*	CGCGCACACCAAGGAGATT
si*‐Cavin1*	CCGCUGUCUACAAGGUGCCGCCUUU

For overexpression, *Cirp*, *Cav1* and *Cavin1* cDNAs were cloned into pCMV6 tagged with Myc and DDK (Origene), and verified by Sanger sequencing. The constructs were transfected into rPAECs with Lipofectamine 3000 (Invitrogen).

### 2.4 Monocrotaline pyrrole (MCTP) treatment

MCTP was extracted and purified in accordance with protocol provided by Mattocks et al^12^ and stored in N,N‐dimethylformamide (DMF) as 100 mg/mL stock solution at −80°C. 48 hours after siRNAs or plasmids transfection, cells were treated with MCTP (50 µg/mL) or equal volume of DMF (vehicle control), and samples were collected after 12 hours for permeability assessments; and after 48 hours for lactate dehydrogenase (LDH) release assay, terminal deoxynucleotidyl transferase (TdT)‐mediated dUTP nick end labelling (TUNEL)‐assay and protein extraction.

### 2.5 LDH release assay

The medium of cultured rPAECs was harvested and centrifuged. The supernatant was collected and incubated with LDH release regents (Beyotime) at 37°C for 1 hour, then mixed with the LDH reaction mixture. The absorbance was recorded at 490 nm wavelength on plate spectrophotometer.

### 2.6 5‐ethynyl‐2’‐deoxyuridine (EdU) assay

To test the effects of rPAECs‐conditioned medium on rPASMCs proliferation, rPAECs were transfected with specific siRNA or plasmids and treated with MCTP for 48 hours. rPASMCs were subjected to 24 hours of growth arrest in serum‐free medium, then treated with conditioned medium from rPAECs. After 48 hours, rPASMCs were fixed for EdU staining in accordance with manufacture's instruction (Thermo).

### 2.7 Permeability assay

Rats were injected 1% evans blue (EB) dye (Sigma) (6 mL/kg) in PBS/4% bovine serum albumin (BSA) via tail vein. One hour later, animals were sacrificed and the lungs were dissected after completely flushing out residual intravascular dye with PBS. Dissected lungs were photographed and homogenized in formamide (Sigma), then incubated at 37°C for 24 hours. After centrifuge, the supernatant was collected and measured at 620 nm on plate spectrophotometer.

To assess endothelial monolayer permeability, transwells (0.4 µm; 24‐well format; Corning) were plated with rPAECs at the density of 5 × 10^3^/well. After transfecting rPAECs with siRNA or plasmids and subsequent MCTP or vehicle treatments, FITC‐dextran (Invitrogen) was added to the upper chamber and incubated at 37°C for 1 hour. Medium (20 µL) was collected from the lower chamber and measured with excitation at 488 nm and emission at 520 nm on plate spectrophotometer.

### 2.8 TUNEL assay

After blocking with PBS/6% goat serum, the lung sections (4 µm) and rPAECs were incubated with TUNEL reaction mixture (Roche) at 37°C in humid box for 1 hour in the dark. Nuclei were stained with DAPI (Sigma). The images were captured with fluorescence microscope (Leica).

### 2.9 RNA immunoprecipitation (RIP)

RNA‐IP was performed according to the Magna RIP RNA‐binding Protein Immunoprecipitation Kit protocol (Millipore). Briefly, rPAECs at 80%‐90% confluency in culture dishes were lysed in RIP lysis buffer, and immunoprecipitated with anti‐CIRP antibody (Proteintech) or normal rabbit IgG (Millipore) conjugated on magnetic beads, then incubated with proteinase K (Millipore) to isolate the immunoprecipitated RNA. Finally, CIRP‐binding RNAs were extracted by acid‐phenol‐chloroform and analysed by qPCR. The primers used are listed in Table 2.

**TABLE 2:** Primers used in the article

Primers	Forward 5’‐3’	Reverse 5’‐3’
Rat‐*Cirp*	GGGTCCTACAGAGACAGCTACGA	CTGGACGCAGAGGGCTTTTA
Rat‐*18s*	AACGAACGAGACTCTGGCATG	CGGACATCTAAGGGCATCACA
Rat‐*Cav1*	CTACAAGCCCAACAACAAGGC	AGGAAGCTCTTGATGCACGGT
Rat‐*Cavin1*	CGGCCAGATAAAGAAACTGG	CCGGCAGCTTGACTTCAT
Rat‐*Nppa*	CAACACAGATCTGATGGATTTCA	CCTCATCTTCTACCGGCATC
Rat‐*Myh7*	GCGGACATTGCCGAGTCCCAG	GCTCCAGGTCTCAGGGCTTCACA

### 2.10 Western blotting

Total protein was extracted with RIPA buffer containing proteinase inhibitor (Roche) and quantified with BCA protein assay reagent (Beyotime), then denatured at 95°C. Proteins were separated on 10% SDS‐polyacrylamide gels and transferred to Immobilon‐P polyvinylidene fluoride (PVDF) membranes. The membranes were blocked with TBS/5% non‐fat milk, then incubated with the following primary antibodies against CAV1 (Abcam), CAVIN1 (Abcam), CIRP (Proteintech) and β‐ACTIN (Santa Cruz) at 4°C overnight and probed with secondary antibodies at room temperature for 1 hour. The signals were detected on Odyssey imager.

### 2.11 qPCR

Total RNA was extracted with Trizol reagent (Invitrogen) according to the manufacturer's instructions, and quantified by Nanodrop 3000. cDNA was synthesized with PrimeScript RT reagent Kit (Takara). qPCR was performed with SYBR Green master mix (Applied Biosystems) as follows: 95°C for 10 minutes, 40 cycles of 95°C for 5 seconds, 60°C for 1 minute and 72°C for 45 seconds. Relative mRNA expression of genes was determined by the 2^−ΔΔCt^ method. The primers used are listed in Table 2.

### 2.12 Protein mass spectrometry

Four weeks after MCT injection, left lungs of *Cirp*‐KO and WT rats were digested into peptides and dissolved in triethylammonium bicarbonate (TEAB). Subsequently, the peptides were incubated with Tandem Mass Tag (TMT) 6‐plex mass tagging reagents, separated on 1100 HPLC System (Agilent) using an Agilent Zorbax Extend RP column (5 μm, 150 mm × 2.1 mm). ProteomeDiscoverer (v.2.2) was used to search all of the Q Exactive raw data thoroughly against Uniport rat database. A global false discovery rate (FDR) was set to 0.01. 1.5‐fold change was defined as the threshold for a significant change in expression.

### 2.13 Statistics

All data shown are the mean ± SD from three independent experiments. Statistical analyses were conducted by Student's *t* test, One‐way ANOVA and Tukey‐Kramer multiple comparisons test.

## 3 RESULTS

### 3.1 *Cirp* was down‐regulated in the lung of MCT‐induced PAH rat

**FIGURE 1:**
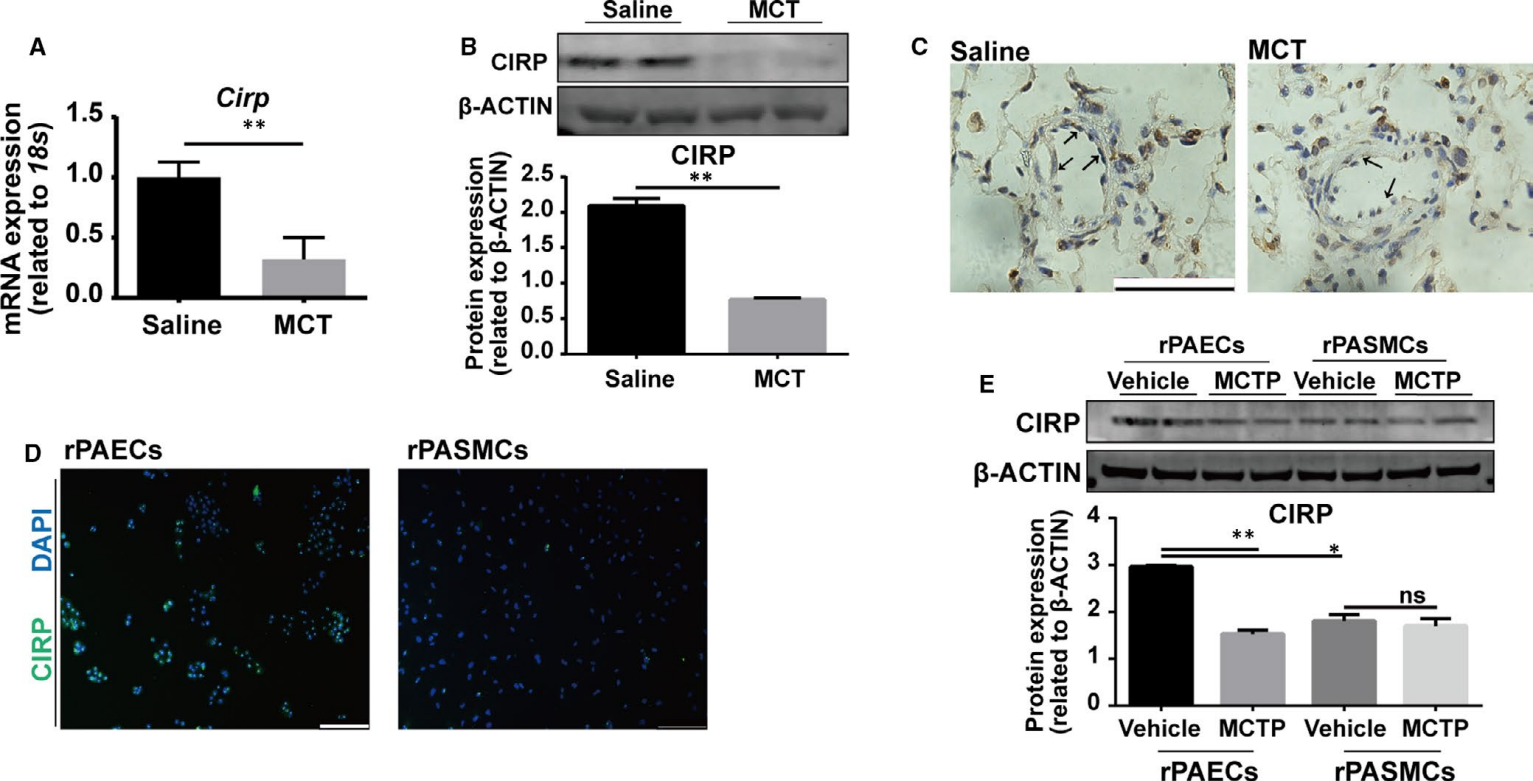
Reduced CIRP expression in the pulmonary artery endothelium of MCT‐induced PAH rats. A, qPCR analysis of *Cirp* expression in rat lungs at week four after saline‐ or MCT‐ injection. (n = 4 per group). B, Western blotting and quantitation of CIRP expression in rat lungs at week four after saline or MCT injection. β‐ACTIN served as loading control. (n = 3 per group). C, Immunofluorescence of CIRP in rat lungs at week four after saline or MCT injection. Black arrows point to CIRP positive signals in endothelium. Scale bar, 50 µm. D, Immunofluorescence of CIRP in rPAECs and rPASMCs. DAPI stains nuclei. Scale bar, 100 µm. E, Western blotting and quantitation of CIRP in rPAECs and rPASMCs following 48 h culture with or without MCTP. β‐ACTIN served as loading control. (n = 3 per group). DAPI, 4′,6‐diamidino‐2‐phenylindole; MCT, Monocrotaline; MCTP, Monocrotaline Pyrrole. The data are shown as mean ± SD from three independent experiments. **P <.01, *P <.05. ns, not significant

To evaluate the expression of *Cirp* in PAH, we established rat PAH model by intraperitoneal injection of MCT. Remarkably, at week four, both mRNA and protein level of CIRP were significantly decreased in the lung of PAH rats revealed by qPCR and Western blotting, respectively (Figure 1A,B). By immunohistochemistry, we found CIRP was widely expressed in the lung, and notably, the reduced expression of CIRP upon MCT administration predominantly localized in the endothelium of pulmonary artery (Figure 1C), indicating the potential role of CIRP in endothelial cell function. To further explore the possible mechanism of CIRP in PAH, we analysed CIRP expression in cultured rPAECs and rPASMCs respectively by immunofluorescence and Western blotting, which detected significantly higher expression of CIRP in rPAECs than in rPASMCs (Figure 1D,E). As MCTP is the active metabolite of MCT in vivo, we treated both rPAECs and rPASMCs with MCTP. Upon MCTP stimulation, the protein level of CIRP was down‐regulated in rPAECs, but barely changed in rPASMCs (Figure 1E), suggesting endothelial cells are the main target of MCTP, where the reduced CIRP expression contributes to MCT‐induced PAH.

### 3.2 *Cirp* deletion exacerbated MCT‐induced PAH

Next, to investigate the role of CIRP in PAH, we generated *Cirp*‐KO rats with seven bases deletion in exon three by transcription activator‐like effector nuclease (TALEN). The *Cirp* gene deletion in *Cirp*‐KO rats was confirmed by Western blotting, in which CIRP protein was barely detected in the lung (Figure S1A). Both *Cirp*‐KO and WT rats were normal in appearance and behaviour.

**FIGURE 2:**
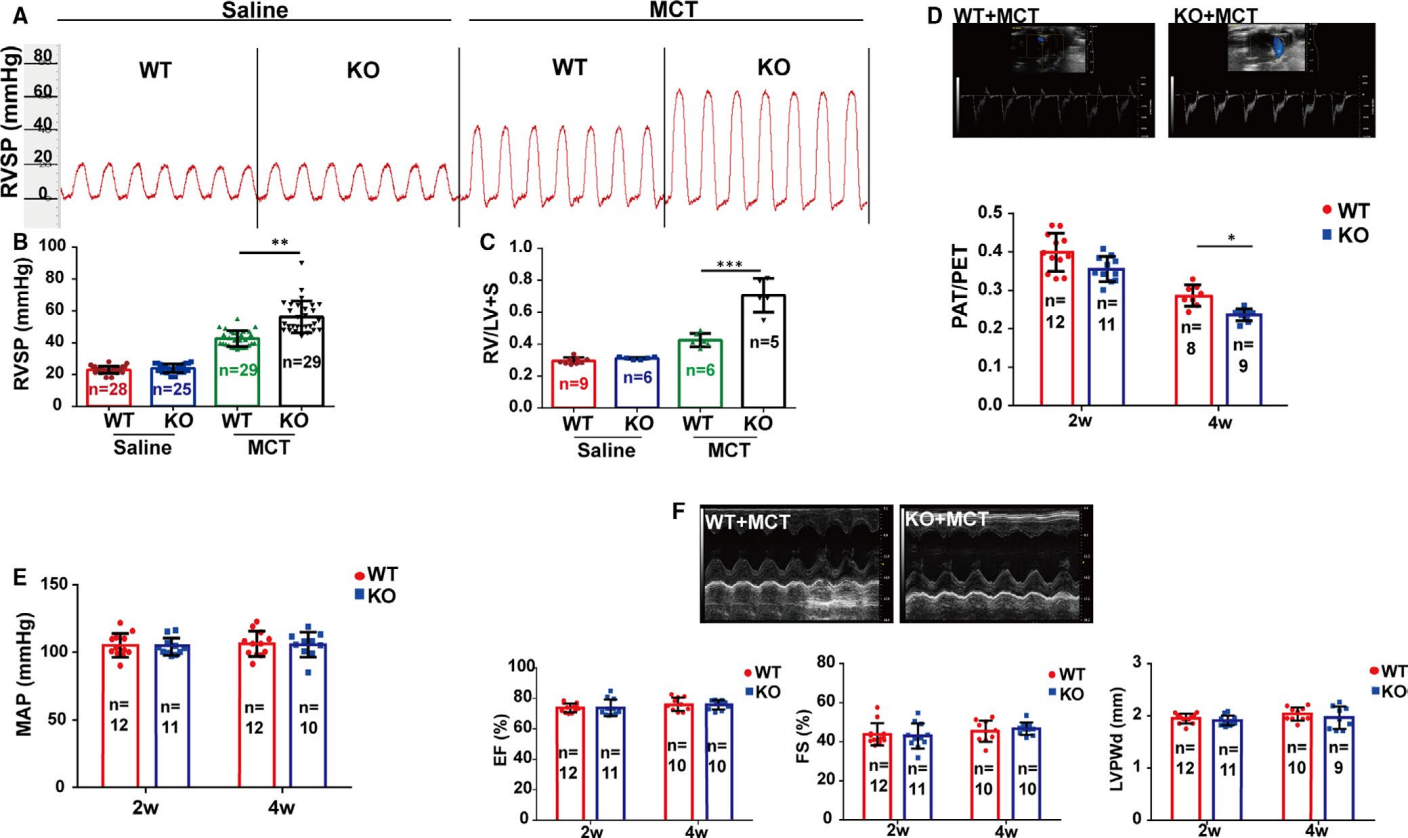
CIRP deficiency exaggerated MCT‐induced PAH in rats. A and B, Right ventricular systolic pressure (RVSP) was assessed by invasive haemodynamic measurements. C, Right ventricular hypertrophy (Fulton index) was evaluated by the right ventricle over the left ventricle + septum weight ratio (RV/(LV + S)). D, Doppler echocardiology was performed to measure the pulmonary artery acceleration time (PAT) and pulmonary artery ejection time (PET) of *Cirp*‐KO and WT rats at week two and four after MCT injection. E, The systole and diastole blood pressure of rats in quiet environment was measured noninvasively to calculate mean artery pressure (MAP). F, Transthoracic two‐dimensional echocardiography was applied on rats to evaluate left ventricular function. Top: representative echocardiographic images of WT and *Cirp*‐KO rats at week four after saline or MCT injection. Bottom: fractional shortening (FS) and the ejection fraction (EF) of left ventricle, left ventricular posterior wall at diastole (LVPWd) were measured on the M‐mode echocardiogram and calculated with standard formula. WT, wild type; KO, *Cirp*‐knockout; MCT, monocrotaline. The data are shown as mean ± SD from three independent experiments. ***P <.001, **P <.01, *P <.05

Although there was no difference in RVSP and RV/LV + S between *Cirp*‐KO and WT rats at baseline (Figure 2A‐C), four weeks after exposure to MCT, higher RVSP was recorded in *Cirp*‐KO rats compared to WT controls (Figure 2A,B). Meanwhile, *Cirp*‐KO rats showed more aggravated right ventricular hypertrophy with elevated RV/LV + S (Figure 2C) as well as increased *Nppa* and *Myh7* expression in right ventricle (Figure S1B,C). Furthermore, the PAT/PET significantly declined in MCT‐treated *Cirp*‐KO rats measured by colour Doppler echocardiography (Figure 2D), suggesting CIRP may function as a protection factor in MCT‐induced PAH.

In our previous research, *Cirp*‐KO rats presented arrhythmia at baseline.^11^ We wonder whether the effect of CIRP on MCT‐induced PAH is partially resulting from the mis‐regulation of system pressure or left cardiac ventricular dysfunction. Thus, we analysed the contribution of system pressure to MCT‐induced PAH by measuring MAP. Notably, no difference could be seen between WT and *Cirp*‐KO rats after MCT treatment (Figure 2E). Moreover, left ventricular function, accessed by M‐mode echocardiography, exhibited no difference in EF, FS and left ventricular posterior wall at diastole (LVPWd) between *Cirp*‐KO and WT rats at week four after MCT injection (Figure 2F). These results excluded the influence of MAP and cardiac function on MCT‐induced PAH both in WT and *Cirp*‐KO rats.

CIRP was widely identified as stress‐response protein which plays pro‐inflammatory role in various diseases,^13^ and MCT could induce significant perivascular mononuclear inflammation in rat lungs.^14^ Thus, the release of CIRP into plasma upon MCT administration may trigger inflammatory response in the lung during the progress of PAH. However, although *Cirp*‐KO rats exhibited exacerbated PAH than WT controls, less infiltration of mononuclear cells in lungs was detected (as evaluated with anti‐CD68 antibody staining, Figure S1D), which indicated inflammatory response is not one of the main factors that contribute to aggravated MCT‐induced PAH in *Cirp*‐KO rats.

### 3.3 Identification of *Cav1* and *Cavin1* as CIRP’s targets in PAH regulation

**FIGURE 3:**
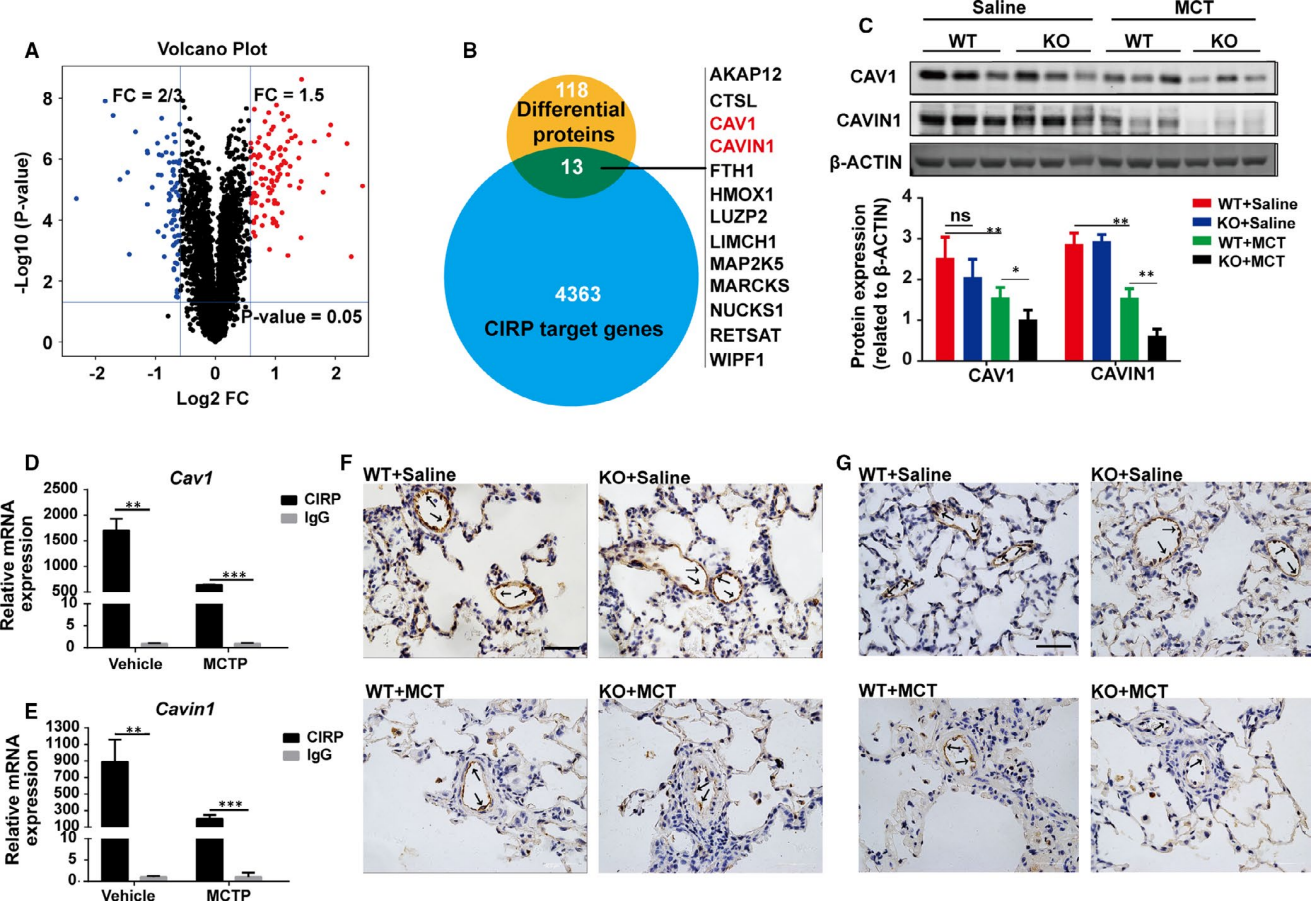
*Cav1* and *Cavin1* served as downstream targets of CIRP in regulating PAH. A, Proteomics was performed in lungs from WT and *Cirp*‐KO rats at week four after saline or MCT injection. (n = 3 per group). The volcano plot illustrated 131 proteins with significant difference between the two groups. B, Potential regulatory molecules were obtained by intersecting published CIRP targeted genes and protein spectrum conducted in this project. Blue circle represents 4376 CIRP target genes identified by PAR‐Clip and RNA sequencing; yellow circle represents 131 differentially expressed proteins in mass spectrometry data. C, Representative immunoblots and densitometric analysis of CAV1 and CAVIN1 in lungs of WT and *Cirp*‐KO rats at week four after saline or MCT injection. β‐ACTIN served as loading control. (n = 3 per group). D and E, The binding of CIRP to *Cav1* (D) and *Cavin1* (E) mRNAs in rPAECs with or without MCTP stimulation was determined by RIP. (n = 3 per group). F and G, Representative immunohistochemical images of lungs in WT and *Cirp*‐KO rats stained for CAV1 (F) and CAVIN1 (G) at week four after saline or MCT injection. Scale bar, 50 µm. WT, wild type; KO, *Cirp*‐knockout; MCT, monocrotaline. The data are shown as mean ± SD from three independent experiments. ***P <.001, **P <.01, *P <.05, ns, not significant

We then set to investigate the underline targets of CIRP in MCT‐induced PAH. By application of protein mass spectrometry, we identified 131 differentially expressed proteins in lungs between *Cirp*‐KO and WT rats four weeks after MCT treatment, including 71 down‐regulated and 60 up‐regulated proteins. (Figure 3A). A key function of CIRP is to bind mRNAs of target genes so as to transcriptionally regulate their expression.^15^ Liu *et al* have reported the overall CIRP targeted mRNAs using photoactivatable ribonucleoside enhanced‐crosslinking and immunoprecipitation (PAR‐Clip) and RNA sequencing in mouse embryonic fibroblasts (MEFs).^16^ By intersecting the published CIRP targeted genes and protein spectrum conducted in this project, we obtained 13 potential targets of CIRP in the lung of rat MCT‐induced PAH (Figure 3B). These 13 genes were all expressed with significant difference in lungs between *Cirp*‐KO and WT rats four weeks after MCT injection. Among the 13 genes, *Cav1* and *Cavin1* attracted our attention, both of which predominantly expressed in pulmonary artery endothelium and were involved in the progress of PAH.^17^ It is worth noting that endothelial cell‐specific knockout of *Cav1* or *Cavin1* respectively could directly induce PAH in C57 mice,^18^, ^19^ and *CAV1* is a well‐known mutant gene in idiopathic pulmonary artery hypertension (IPAH) patients.^20^ We performed RIP on rPAECs, which revealed dramatic enrichment of CIRP on both *Cav1* and *Cavin1* mRNAs, verifying these two genes as direct targets of CIRP (Figure 3D,E). Furthermore, the binding of CIRP to either *Cav1* or *Cavin1* was significantly reduced after MCTP stimulation (Figure 3D,E).

We then analysed the expression of CAV1 and CAVIN1 in the lungs of *Cirp*‐KO rats four weeks after MCT treatment. Compared with WT rats, significantly lower amount of both CAV1 and CAVIN1 mRNA (Figure S2A,B) and protein (Figure 3C) was detected in *Cirp*‐KO rats. By immunohistochemistry, both CAV1 and CAVIN1 mainly expressed in pulmonary artery endothelium, and decreased drastically after MCT treatment especially in CIRP deficiency ones (Figure 3F,G). These data suggest that CAV1 and CAVIN1 may mediate CIRP in maintaining homeostasis of endothelium.

### 3.4 CIRP regulated MCTP‐induced rPAECs apoptosis and permeability through CAV1 and CAVIN1

**FIGURE 4:**
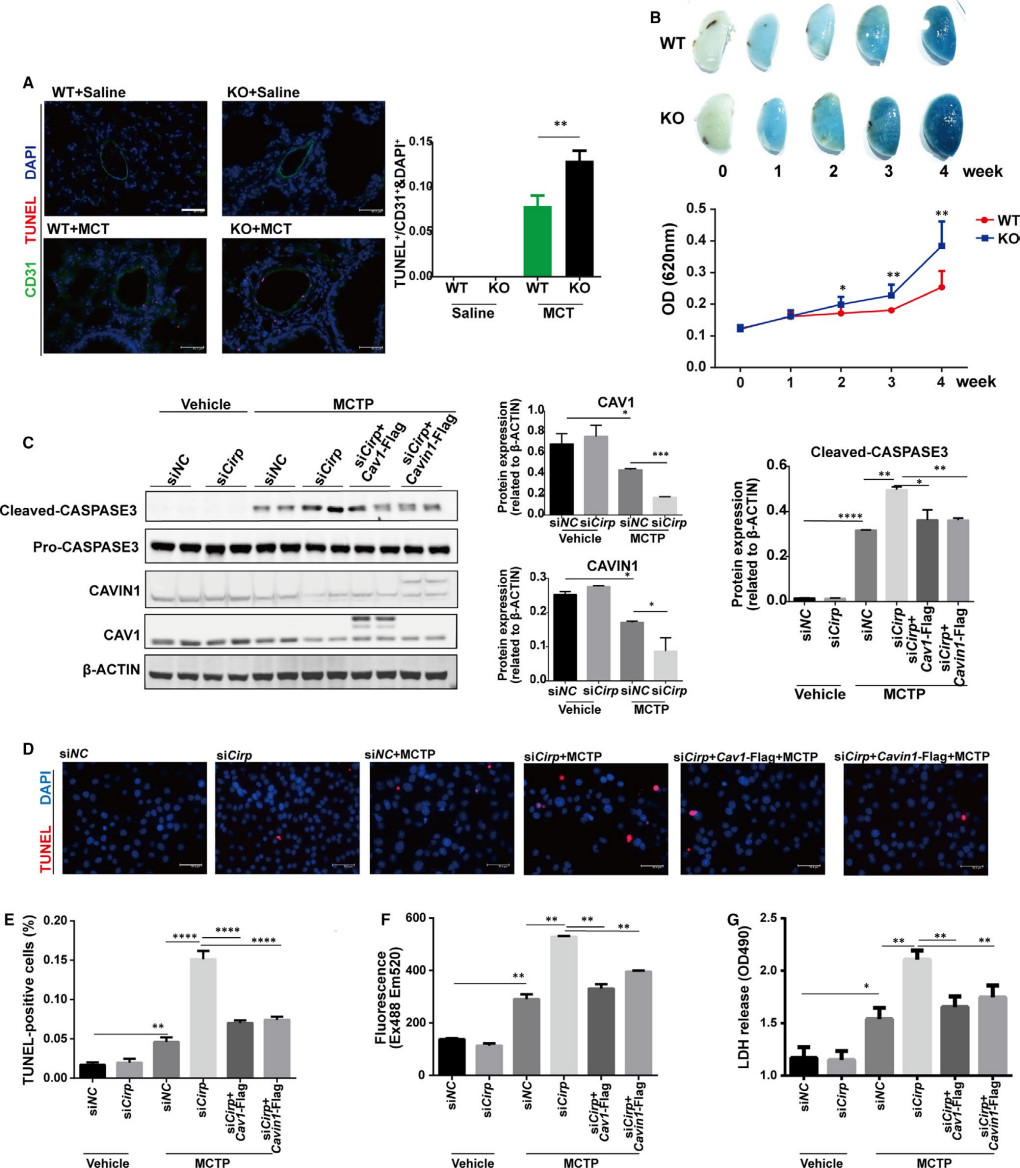
*Cirp* knockdown exacerbated MCTP‐induced rPAECs apoptosis and permeability that was counteracted by *Cav1* or *Cavin1* overexpression. A, TUNEL assay of lungs isolated from WT and *Cirp*‐KO rats at week four after saline or MCT injection. The apoptotic index was measured by counting puncta of TUNEL signal in randomly selected CD31‐positive cells (n ≥ 3 per group). Scale bar, 46 µm. B, Images of EB dye staining in lungs isolated from WT and *Cirp*‐KO rats at different time points after MCT injection respectively, and quantitative assessment of EB dye content in the lungs. C, Representative immunoblots and densitometric analysis of cleaved CASPASE‐3, CAV1 and CAVIN1 in *Cirp* knockdown and *Cav1* or *Cavin1* overexpressing rPAECs following MCTP treatment. β‐ACTIN served as loading control. (n = 3 per group). D, TUNEL assay of *Cirp* knockdown and *Cav1* or *Cavin1* overexpressing rPAECs following MCTP treatment. DAPI stains nuclei. Scale bars, 50.4 µm. E, The apoptotic index was measured by counting puncta of TUNEL positive signal. F and G, Permeability and cytotoxicity of *Cirp* knockdown and *Cav1* or *Cavin1* overexpressing rPAECs following MCTP treatment were assessed by quantifying absorbance of FITC‐dextran (F), LDH release (G). WT, wild type; KO, *Cirp*‐knockout; MCT, monocrotaline; MCTP, monocrotaline pyrrole; TUNEL, Terminal deoxynucleotidyl transferase‐mediated dUTP‐biotin nick end labelling; LDH, lactate dehydrogenase; CD31, Platelet endothelial cell adhesion molecule‐1, PECAM‐1/CD31; DAPI, 4′,6‐diamidino‐2‐phenylindole; The data are shown as mean ± SD from three independent experiments. **** *P* <.0001, ***P <.001, ** *P* <.01, * *P* <.05. ns, not significant

Since MCTP directly targets endothelium in vivo and induces excessive PAECs apoptosis, which is detrimental to vascular function, endothelium is considered to be the primary target in this process, eventually leading to PAH.^21^, ^22^, ^23^ By TUNEL assay, we found *Cirp* depletion indeed aggravated rPAECs apoptosis in response to MCT treatment in vivo (Figure 4A). Meanwhile, in cultured rPAECs, MCTP‐induced pronounced apoptosis indicated by increased cleaved CASPASE 3, which was intensified by *Cirp* knockdown (Figure S3A) and counteracted by *Cirp* overexpression (Figure S3B).

One aspect of endothelium dysfunction contributing to PAH is the continuous damage of PAECs, resulting in increased vessel permeability.^24^ We therefore injected EB dye via tail vein to assess the endothelium integrity in vivo. Though no vessel leakage manifested in both unstimulated WT and *Cirp*‐KO rats (Figure 4B), elevated extravasation of EB dye into lungs was detected following MCT injection with *Cirp*‐KO rats presenting much higher permeability two weeks after MCT treatment (Figure 4B). These results indicated that CIRP deficiency deteriorated MCT‐induced endothelium damage.

Based on the previous data that *Cav1* and *Cavin1* may be downstream targets of CIRP in MCT‐induced PAH, we then analysed their expression when up‐ or down‐ regulating *Cirp* in cultured rPAECs with or without MCTP treatment. Consistent with their in vivo expression, CAV1 and CAVIN1 were both down‐regulated in MCTP‐treated rPAECs, particularly when *Cirp* was knockdown (Figure 4C). On the contrary, *Cirp* overexpression reversed the reduction of CAV1 and CAVIN1 in the presence of MCTP (Figure S4A). Furthermore, as assessed by TUNEL assay and cleaved‐CASPASE3 expression, overexpression of *Cav1* or *Cavin1* inhibited the increase in apoptosis caused by *Cirp* knockdown in MCTP‐treated rPAECs (Figure 4C‐E). On the other hand, down‐regulation of *Cav1* or *Cavin1* cancelled the protection of rPAECs from MCTP‐induced apoptosis by *Cirp* overexpression (Figure S4A‐C). Therefore, CIRP deficiency intensified MCTP‐induced rPAECs apoptosis both in vivo and in vitro, which contributes significantly to the pathogenesis of PAH. And the function of CIRP in MCT‐induced PAH is partially through regulating CAV1 and CAVIN1.

Besides apoptosis, MCTP‐induced increased vessel permeability in vivo. We cultured rPAECs in monolayer and analysed its permeability with FITC‐dextran leakage assay. While knockdown of *Cirp* displayed increased rPAEC permeability after MCTP treatment which was partially rescued by *Cav1* or *Cavin1* overexpression (Figure 4F), *Cirp* overexpression preserved the integrity of monolayer, which was disrupted by *Cav1* or *Cavin1* knockdown (Figure S4D). Meanwhile, LDH release assay also verified that reduced *Cirp* accelerates MCTP‐induced rPAECs injury, while *Cirp* overexpression showed protecting effects, both of which could be counteracted by *Cav1*/*Cavin1* up‐ (Figure 4G) or down‐ (Figure S4E) regulation respectively.

### 3.5 CIRP regulated the proliferative effect of conditioned media from MCTP‐treated rPAECs on rPASMCs

**FIGURE 5:**
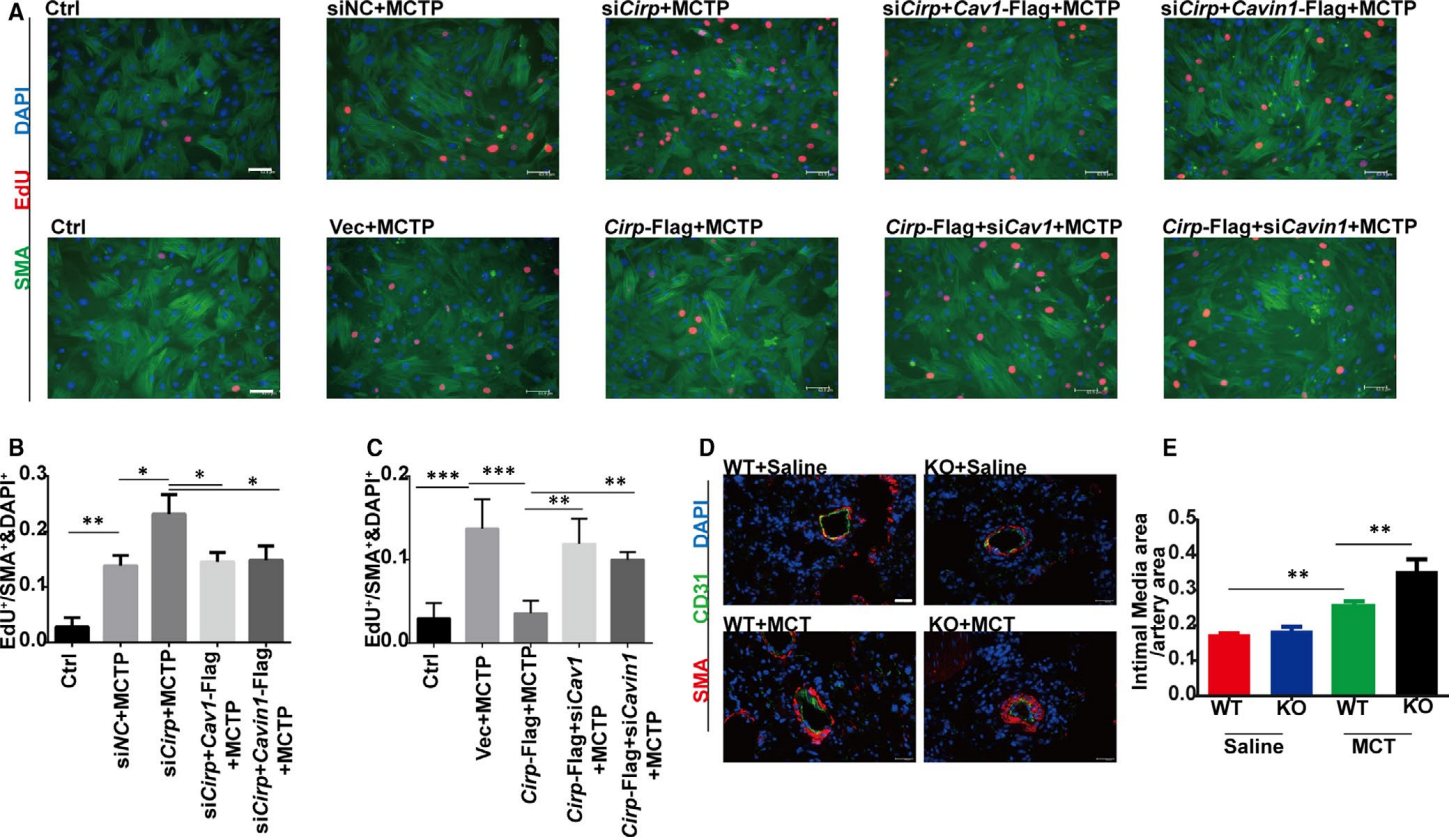
Effects of conditioned media from MCTP‐treated rPAECs on rPASMCs proliferation. A, EdU staining of rPASMCs cultured in conditioned media from rPAECs. Top: conditioned media from *Cirp* knockdown and *Cav1* or *Cavin1* overexpressing rPAECs following MCTP treatment. Bottom: conditioned media from *Cirp* overexpressing and *Cav1* or *Cavin1* knockdown. DAPI stains nuclei. Scale bar, 63.9 µm. B and C, The proliferate index was measured by counting puncta of EdU signal. (n ≥ 3 per group). D, Immunofluorescence of SMA and CD31 in the lungs of WT and KO rats at week four after saline or MCT injection. DAPI stains nuclei. Scale bar, 100 µm. E, The thickness of intimal media was determined by intimal media area over pulmonary artery area of WT and *Cirp*‐KO rats at week four after saline or MCT injection. (n ≥ 3 per group). WT, wild type; KO, *Cirp*‐knockout; MCT, monocrotaline; EdU, 5‐ethynyl‐2’‐deoxyuridine; SMA, smooth muscle actin; CD31, platelet endothelial cell adhesion molecule‐1, PECAM‐1/CD31; DAPI, 4′,6‐diamidino‐2‐phenylindole. The data are shown as mean ± SD from three independent experiments. *** *P* <.001, ** *P* <.01, * *P* <.05

Although the regulatory function of CIRP in MCT‐induced PAH is mainly by protecting endothelium from injury, we also observed exceedingly thickened intimal media of pulmonary artery in *Cirp*‐KO rats (Figure 5D,E). In PAH, the damaged endothelium could secret growth factors and vasoactive mediators, which induced intimal media proliferation. To test the effects of damaged rPAECs on rPASMCs, we cultured MCTP‐treated rPAECs, collected the conditioned media and applied it onto rPASMCs. As shown in Figure 5A,B, increased number of EdU^+^ cells were detected suggesting the proliferative effect of MCTP‐treated rPAECs medium. We then transfected rPAECs with *Cirp*‐siRNA or *Cirp*‐Flag before MCTP stimulation. When rPASMCs were cultured in conditioned media from MCTP‐treated rPAECs with *Cirp* knockdown or overexpression, the proliferation of rPASMCs was significantly increased (Figure 5A,B) or decreased (Figure 5A,C), respectively. Moreover, when *Cav1* and *Cavin1* were overexpressed or down‐regulated in *Cirp* knockdown or overexpressing rPAECs, after MCTP treatment, the conditioned media from these cells increased and decreased the EdU^+^ rPASMCs respectively (Figure 5A‐C), again demonstrating CAV1 and CAVIN1 acted as downstream effectors of CIRP. The rPASMCs proliferative factors secreted by rPAECs warrant further investigation.

## 4 DISCUSSION

In this study, *Cirp*‐KO rats showed aggravated endothelium damage in MCT‐induced PAH. Protein mass spectrometry analysis provided evidence that CAV1 and CAVIN1 are potential targets of CIRP in this process. In subsequent functional analysis, we observed increased apoptosis and hyperpermeability in MCTP‐treated rPAECs with *Cirp* depletion, which was caused by reduced expression of CAV1 or CAVIN1, indicating that CIRP mediates MCT‐induced PAH through regulating the level of CAV1 and CAVIN1 in pulmonary artery endothelium.

Drugs or toxins exposure often causes devastating damage to pulmonary artery endothelium, eventually leading to PAH.^9^, ^25^, ^26^ MCT is an alkaloid that is often used to induce PAH in rodents, which could mimic a great deal of pathophysiological features in human PAH.^27^ It has been reported that MCTP induces LDH release and apoptosis of endothelial cells.^22^ Although rPAECs manifested increased apoptosis and hyperpermeability when treated with MCTP,^22^, ^28^ rPASMCs presented minimal changes.^21^ Thus, in MCT‐induced PAH, endothelium is widely recognized as the primary target of MCTP. Our data demonstrated the profound role of rPAECs in CIRP’s regulation of MCT‐induced PAH.

At present, reduced CIRP expression was reported when cells were exposed to heat stress and hypoxia *etc*, indicating that CIRP served as a protective factor against stressful conditions.^29^ For the first time, we discovered that CIRP expression decreased, particularly, in endothelium of MCTP‐induced PAH, and genetically depletion of *Cirp* aggravated endothelium apoptosis and permeability, consistent with its protective role in other stress and diseases. Knockdown of *Cirp* could promote apoptosis in various cells in different disease models. For instance, knocking down *Cirp* could aggravate apoptosis in cardiomyocytes.^30^ Our data demonstrated that down‐regulation of *Cirp* promoted MCTP‐induced rPAECs apoptosis, which was reversed by *Cirp* overexpression. MCT causes progressive endothelial cell membrane damage starting from 24 to 48 hours post‐injection.^31^, ^32^ The causal link between the gradually reduced CIRP expression and progressive endothelial cell membrane damage is unclear. There was time‐dependent loss of CIRP in rat lungs after MCT injection (Figure S5A). Moreover, in cultured rPAECs, MCTP‐induced reduction of CIRP expression was reversed by *Cav1* overexpression (Figure S5B), which partly accounts for the possibility that the loss of CIRP is the result of the progressive endothelial cell membrane damage.

The predominant function of CIRP is to stabilize mRNAs of target genes.^33^ Liu et al showed CIRP binds to 3’ untranslated region (UTR) of *Cav1* and coding sequence (CDS) of *Cavin1,* respectively. Down‐regulation of *Cirp* decreased mRNA expression of *Cav1* and *Cavin1*.^16^ Similarly, our study confirmed the binding of CIRP to *Cav1* and *Cavin1* mRNAs in rPAECs, which was significantly reduced after MCTP stimulation. Interestingly, we found that in the absence of MCT, the *Cav1* and *Cavin1* mRNAs in *Cirp*‐KO rats were less than those of WT rats (Figure S2A,B); however, at protein level, little difference was observed. The protein abundance regulation credibly reflects biological roles of specific genes. However, approximately one‐ to two‐thirds of the variance in steady‐state protein level can be explained by partial correlation of mRNA transcript and protein abundances.^34^ The drift of mRNA transcript and protein abundance may be compensated by translational and protein‐degradational regulation.^34^ Thus, we speculated that CIRP acted as one of stabilizers of *Cav1* or *Cavin1* in endothelium, which was disrupted upon MCTP stimulation. Although the regulators of CAV1 and CAVIN1 in endothelium are not just CIRP, the deficiency of CIRP may lead to CAV1 or CAVIN1 more likely to lose upon pathological insults. The role of CIRP in maintaining homeostasis of CAV1 or CAVIN1 warrants further investigation.

Core caveola structural proteins CAV1 and CAVIN1 are widely expressed in various cell types and predominantly enriched in endothelial cells,^35^ both of which are essential for constituting and maintaining structure and function of caveolae.^17^ Frame‐shift mutation of *CAV1* is common in IPAH, which causes reduced caveolae number and disfunction.^36^ In MCT‐treated rat lung, the expression of CAV1 mainly decreased in artery endothelium rather than venous endothelium,^32^ resulting in about 25% reduction of caveolae per unit endothelial cell volume.^37^ Moreover, *Cav1* or *Cavin1* knockout mice presented hyperplasia in PAECs and high incidence of spontaneous PAH,^18^, ^19^ suggesting that the number and function of caveolae are critical in maintaining endothelium homeostasis. In our MCT‐induced rat PAH model, the expression of CAV1 and CAVIN1 significantly decreased along with the reduction of CIRP in endothelium. These results uniformly provided evidence that endothelial cells are the main target in this disease model, indicating the potential role of CIRP in caveola biology through regulating CAV1 and CAVIN1 expression. Nevertheless, we did not clarify how CIRP binds to *Cav1* and *Cavin1* mRNA and regulates their post‐transcriptional expression, which will be the direction of our following researches.

Caveolae are unique plasma membrane microdomains which regulate endothelial cell permeability through endocytosis of macromolecules.^38^ The reduction of caveolae in IPAH may result in hyperpermeability in pulmonary endothelium and subsequent vascular damage.^39^ Moreover, knockout of *Cav1* exhibited smaller endothelial cell tight junctions, which disrupted the integrity of endothelium.^40^ *Cirp*‐KO rats manifested more EB dye leakage in the lung after MCT treatment, indicating more serious damage to endothelium. In rPAECs, RIP revealed the binding of CIRP to both *Cav1* and *Cavin1* mRNAs, which was greatly reduced after MCTP stimulation. Moreover, *Cirp* knockdown enhanced the permeability of FITC‐dextran and release of LDH, which could be inhibited by *Cav1* or *Cavin1* overexpression, further confirming the functional role of CAV1 and CAVIN1 in CIRP‐dependent endothelial cell apoptosis and permeability, suggesting CAV1 and CAVIN1 are downstream targets of CIRP in PAH.

In response to stimulation, damaged endothelial cells in PAH could release various vasomediators and growth factors inducing vasoconstriction and smooth muscle cell proliferation and eventually leading to pulmonary vascular luminal obliteration and resistance.^41^ From the results obtained in this research, we assumed that MCTP could induce rPAECs to secret factors to promote rPASMCs proliferation, which is fundamental to pulmonary vascular remodelling. As the proliferative effect of conditioned media was also correlated to CIRP, CAV1 and CAVIN1 expression in MCTP‐treated rPAECs, it is intriguing to identify these proliferation factors and investigate the underlying mechanisms, that is, how CIRP regulates their expression, which may facilitate the development of new targets for the treatment of PAH.

The loss of CIRP aggravated endothelial injury by down‐regulating CAV1 and CAVIN1, which are essential in maintaining endothelium homeostasis. The protective role of CIRP in MCT‐induced endothelium damage indicated that overexpression of CIRP in endothelium may serve as a protective factor in toxin‐induced PAH. In the future, gene therapy targeting CIRP may become a novel intervention against PAH in clinical practice. However, because CIRP is widely expressed in tissues and organs, the molecular mechanisms that CIRP regulates CAV1 and CAVIN1 in endothelium need further investigation in order to circumvent any possible side effects.

To summarize, we characterized CIRP’s critical role in MCT‐induced PAH. In *Cirp*‐KO rats, MCT induced aggravated PAH with severe endothelium damage. The main function of CIRP in MCT‐induced PAH is to protect endothelial cells from apoptosis and to maintain endothelium integrity, which is mediated by its downstream targets CAV1 and CAVIN1.

## CONFLICT OF INTEREST

The authors confirm that there are no conflicts of interest.

## AUTHOR CONTRIBUTIONS

**Jingjing Liu:** Data curation (lead); Investigation (lead); Methodology (lead); Validation (lead); Visualization (lead); Writing‐original draft (lead). **Xianting Ke:** Data curation (supporting); Formal analysis (supporting); Investigation (supporting); Methodology (supporting); Supervision (supporting); Validation (supporting); Visualization (supporting). **Luxin Wang:** Data curation (supporting); Formal analysis (supporting); Investigation (supporting); Methodology (supporting); Supervision (supporting); Visualization (supporting). **Yangyang Zhang:** Conceptualization (lead); Data curation (equal); Investigation (supporting); Methodology (equal); Project administration (supporting); Supervision (equal); Validation (equal). **Jian Yang:** Conceptualization (equal); Formal analysis (equal); Funding acquisition (lead); Project administration (lead); Resources (lead); Software (equal); Supervision (lead); Validation (equal); Visualization (equal); Writing‐review & editing (lead).

## Supporting information

Fig S1

Fig S2

Fig S3

Fig S4

Fig S5A‐B

Fig S5B‐wb‐CAV1‐adjusted

Fig S5B‐wb‐CAV1

Fig S5B‐wb‐CIRP

Fig S5B‐wb‐OP¦‐ACTIN

## Data Availability

The data that support the findings of this study are available from the corresponding author upon reasonable request.

## References

[1] Hoeper MM , Humbert M , Souza R , et al. A global view of pulmonary hypertension. Lancet Respir Med. 2016;4(4):306‐322. 26975810 10.1016/S2213-2600(15)00543-3

[2] Kiely DG , Elliot CA , Sabroe I , Condliffe R . Pulmonary hypertension: diagnosis and management. BMJ. 2013;346:f2028. 23592451 10.1136/bmj.f2028

[3] Rabinovitch M . Molecular pathogenesis of pulmonary arterial hypertension. J Clin Investig. 2012;122(12):4306‐4313. 23202738 10.1172/JCI60658 PMC3533531

[4] MacIver DH , Adeniran I , MacIver IR , Revell A , Zhang H . Physiological mechanisms of pulmonary hypertension. Am Heart J. 2016;180:1‐11. 27659877 10.1016/j.ahj.2016.07.003

[5] Alan B , Nalbantgil S . Genetic, cellular and molecular mechanisms of pulmonary arterial hypertension. Anadolu Kardiyoloji Derg/Anatolian J Cardiol. 2010;10(Suppl 1):9‐13. 10.5152/akd.2010.114 20819762

[6] Sommer N , Ghofrani A , Pak O , et al. Current and future treatments of pulmonary arterial hypertension. Br J Pharmacol. 2021;178(1):6‐30. 32034759 10.1111/bph.15016

[7] Li SZ , Jin FH , Zhao QX . Progress of research on CIRP and its biological functions. Sheng li ke xue jin zhan [Progress in physiology]. 2009;40(3):271‐273. 19803437

[8] Zhu X , Buhrer C , Wellmann S . Cold‐inducible proteins CIRP and RBM3, a unique couple with activities far beyond the cold. Cell Mol Life Sci. 2016;73(20):3839‐3859. 27147467 10.1007/s00018-016-2253-7 PMC5021741

[9] Hoorn CM , Wagner JG , Roth RA . Effects of monocrotaline pyrrole on cultured rat pulmonary endothelium. Toxicol Appl Pharmacol. 1993;120(2):281‐287. 8511798 10.1006/taap.1993.1113

[10] Cheng W , Oike M , Hirakawa M , Ohnaka K , Koyama T , Ito Y . Excess l‐arginine restores endothelium‐dependent relaxation impaired by monocrotaline pyrrole. Toxicol Appl Pharmacol. 2005;207(3):187‐194. 16129112 10.1016/j.taap.2005.01.002

[11] Li J , Xie D , Huang J , et al. Cold‐inducible RNA‐binding protein regulates cardiac repolarization by targeting transient outward potassium channels. Circ Res. 2015;116(10):1655‐1659. 25953924 10.1161/CIRCRESAHA.116.306287

[12] Mattocks AR , Jukes R , Brown J . Simple procedures for preparing putative toxic metabolites of pyrrolizidine alkaloids. Toxicon. 1989;27(5):561‐567. 2749755 10.1016/0041-0101(89)90117-7

[13] Zhong P , Peng J , Yuan M , Kong B , Huang H . Cold‐inducible RNA‐binding protein (CIRP) in inflammatory diseases: molecular insights of its associated signalling pathways. Scand J Immunol. 2021;93(1):e12949. 32738154 10.1111/sji.12949

[14] Wilson DW , Segall HJ , Pan LC , Lame MW , Estep JE , Morin D . Mechanisms and pathology of monocrotaline pulmonary toxicity. Crit Rev Toxicol. 1992;22(5–6):307‐325. 1489509 10.3109/10408449209146311

[15] Yang R , Weber DJ , Carrier F . Post‐transcriptional regulation of thioredoxin by the stress inducible heterogenous ribonucleoprotein A18. Nucleic Acids Res. 2006;34(4):1224‐1236. 16513844 10.1093/nar/gkj519 PMC1388095

[16] Liu Y , Hu W , Murakawa Y , et al. Cold‐induced RNA‐binding proteins regulate circadian gene expression by controlling alternative polyadenylation. Sci Rep. 2013;3:2054. 23792593 10.1038/srep02054 PMC3690385

[17] Chettimada S , Yang J , Moon HG , Jin Y . Caveolae, caveolin‐1 and cavin‐1: emerging roles in pulmonary hypertension. World J Respirol. 2015;5(2):126‐134. 28529892 10.5320/wjr.v5.i2.126 PMC5438095

[18] Zhao YY , Liu Y , Stan RV , et al. Defects in caveolin‐1 cause dilated cardiomyopathy and pulmonary hypertension in knockout mice. Proc Natl Acad Sci USA. 2002;99(17):11375‐11380. 12177436 10.1073/pnas.172360799 PMC123264

[19] Sward K , Sadegh MK , Mori M , Erjefalt JS , Rippe C . Elevated pulmonary arterial pressure and altered expression of Ddah1 and Arg1 in mice lacking cavin‐1/PTRF. Physiol Rep. 2013;1(1):e00008. 24303100 10.1002/PHY2.8 PMC3831936

[20] Garcia‐Rivas G , Jerjes‐Sanchez C , Rodriguez D , Garcia‐Pelaez J , Trevino V . A systematic review of genetic mutations in pulmonary arterial hypertension. BMC Med Genet. 2017;18(1):82. 28768485 10.1186/s12881-017-0440-5 PMC5541665

[21] Reindel JF , Roth RA . The effects of monocrotaline pyrrole on cultured bovine pulmonary artery endothelial and smooth muscle cells. Am J Pathol. 1991;138(3):707‐719. 2000943 PMC1886276

[22] Thomas HC , Lame MW , Dunston SK , Segall HJ , Wilson DW . Monocrotaline pyrrole induces apoptosis in pulmonary artery endothelial cells. Toxicol Appl Pharmacol. 1998;151(2):236‐244. 9707500 10.1006/taap.1998.8458

[23] Bueno‐Beti C , Sassi Y , Hajjar RJ , Hadri L . Pulmonary artery hypertension model in rats by monocrotaline administration. Methods Mol Biol. 2018;1816:233‐241. 29987824 10.1007/978-1-4939-8597-5_18

[24] Taylor DW , Lame MW , Nakayama LS , Segall HJ , Wilson DW . Effects of monocrotaline pyrrole and thrombin on pulmonary endothelial cell junction and matrix adhesion proteins. Toxicology. 2003;184(2–3):227‐240. 12499124 10.1016/s0300-483x(02)00582-6

[25] Haworth SG . Role of the endothelium in pulmonary arterial hypertension. Vascul Pharmacol. 2006;45(5):317‐325. 17005453 10.1016/j.vph.2006.08.006

[26] Stacher E , Graham BB , Hunt JM , et al. Modern age pathology of pulmonary arterial hypertension. Am J Respir Crit Care Med. 2012;186(3):261‐272. 22679007 10.1164/rccm.201201-0164OC PMC3886716

[27] Gomez‐Arroyo JG , Farkas L , Alhussaini AA , et al. The monocrotaline model of pulmonary hypertension in perspective. Am J Physiol Lung Cell Mol Physiol. 2012;302(4):L363‐L369. 21964406 10.1152/ajplung.00212.2011

[28] Rafikova O , James J , Eccles CA , et al. Early progression of pulmonary hypertension in the monocrotaline model in males is associated with increased lung permeability. Biol Sex Differ. 2020;11(1):11. 32188512 10.1186/s13293-020-00289-5 PMC7079376

[29] Liao Y , Tong L , Tang L , Wu S . The role of cold‐inducible RNA binding protein in cell stress response. Int J Cancer. 2017;141(11):2164‐2173. 28608439 10.1002/ijc.30833

[30] Zhao HL , Wu BQ , Luo Y , et al. Exogenous hydrogen sulfide ameliorates high glucose‐induced myocardial injury & inflammation via the CIRP‐MAPK signaling pathway in H9c2 cardiac cells. Life Sci. 2018;208:315‐324. 29857073 10.1016/j.lfs.2018.05.051

[31] Schultze AE , Gunaga KP , Wagner JG , Hoorn CM , Moorehead WR , Roth RA . Lactate dehydrogenase activity and isozyme patterns in tissues and bronchoalveolar lavage fluid from rats treated with monocrotaline pyrrole. Toxicol Appl Pharmacol. 1994;126(2):301‐310. 8209383 10.1006/taap.1994.1120

[32] Mathew R , Huang J , Shah M , Patel K , Gewitz M , Sehgal PB . Disruption of endothelial‐cell caveolin‐1alpha/raft scaffolding during development of monocrotaline‐induced pulmonary hypertension. Circulation. 2004;110(11):1499‐1506. 15353500 10.1161/01.CIR.0000141576.39579.23

[33] Xia Z , Zheng X , Zheng H , Liu X , Yang Z , Wang X . Cold‐inducible RNA‐binding protein (CIRP) regulates target mRNA stabilization in the mouse testis. FEBS Lett. 2012;586(19):3299‐3308. 22819822 10.1016/j.febslet.2012.07.004

[34] Vogel C , Marcotte EM . Insights into the regulation of protein abundance from proteomic and transcriptomic analyses. Nat Rev Genet. 2012;13(4):227‐232. 22411467 10.1038/nrg3185 PMC3654667

[35] Sowa G . Caveolae, caveolins, cavins, and endothelial cell function: new insights. Front Physiol. 2012;2:120. 22232608 10.3389/fphys.2011.00120 PMC3252561

[36] Austin ED , Ma L , LeDuc C , et al. Whole exome sequencing to identify a novel gene (caveolin‐1) associated with human pulmonary arterial hypertension. Circ Cardiovasc Genet. 2012;5(3):336‐343. 22474227 10.1161/CIRCGENETICS.111.961888 PMC3380156

[37] Rosenberg HC , Rabinovitch M . Endothelial injury and vascular reactivity in monocrotaline pulmonary hypertension. Am J Physiol. 1988;255(6 Pt 2):H1484‐1491. 3144186 10.1152/ajpheart.1988.255.6.H1484

[38] Sun Y , Minshall RD , Hu G . Role of caveolin‐1 in the regulation of pulmonary endothelial permeability. Methods Mol Biol. 2011;763:303‐317. 21874461 10.1007/978-1-61779-191-8_21

[39] Prewitt AR , Ghose S , Frump AL , et al. Heterozygous null bone morphogenetic protein receptor type 2 mutations promote SRC kinase‐dependent caveolar trafficking defects and endothelial dysfunction in pulmonary arterial hypertension. J Biol Chem. 2015;290(2):960‐971. 25411245 10.1074/jbc.M114.591057 PMC4294523

[40] Schubert W , Frank PG , Woodman SE , et al. Microvascular hyperpermeability in caveolin‐1 (‐/‐) knock‐out mice. Treatment with a specific nitric‐oxide synthase inhibitor, L‐NAME, restores normal microvascular permeability in Cav‐1 null mice. J Biol Chem. 2002;277(42):40091‐40098. 12167625 10.1074/jbc.M205948200

[41] Budhiraja R , Tuder RM , Hassoun PM . Endothelial dysfunction in pulmonary hypertension. Circulation. 2004;109(2):159‐165. 14734504 10.1161/01.CIR.0000102381.57477.50

