# Supplementary figures and images for: Deficiency of cold‐inducible RNA‐binding protein exacerbated monocrotaline‐induced pulmonary artery hypertension through Caveolin1 and CAVIN1
Source: J Cell Mol Med. 2021 Mar 23;25(10):4732–43. doi: 10.1111/jcmm.16437 (PMC8107102; doi:10.1111/jcmm.16437)

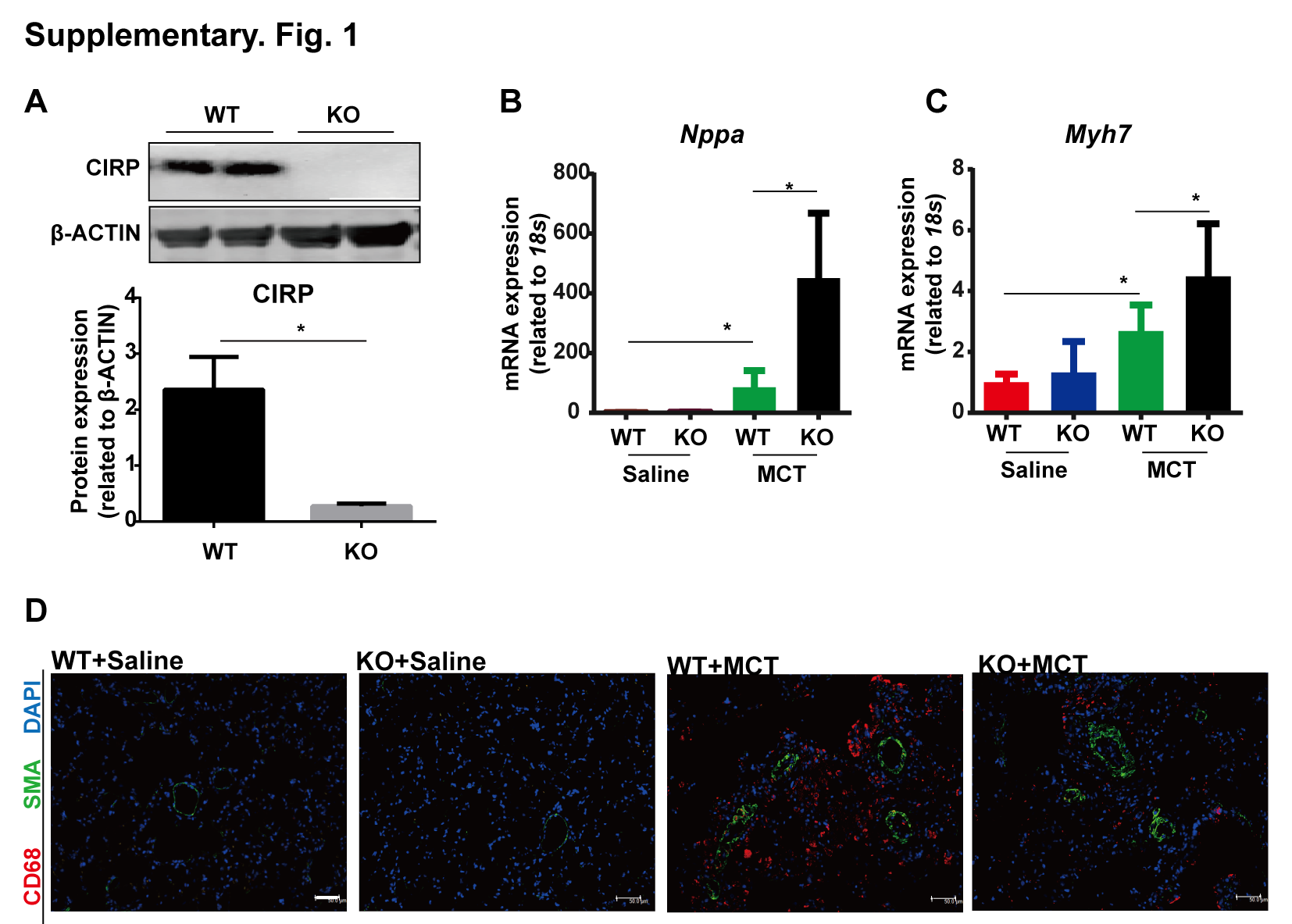

Supplement: Supplementary file 1 — Fig S1 [file JCMM-25-4732-s006.tif]

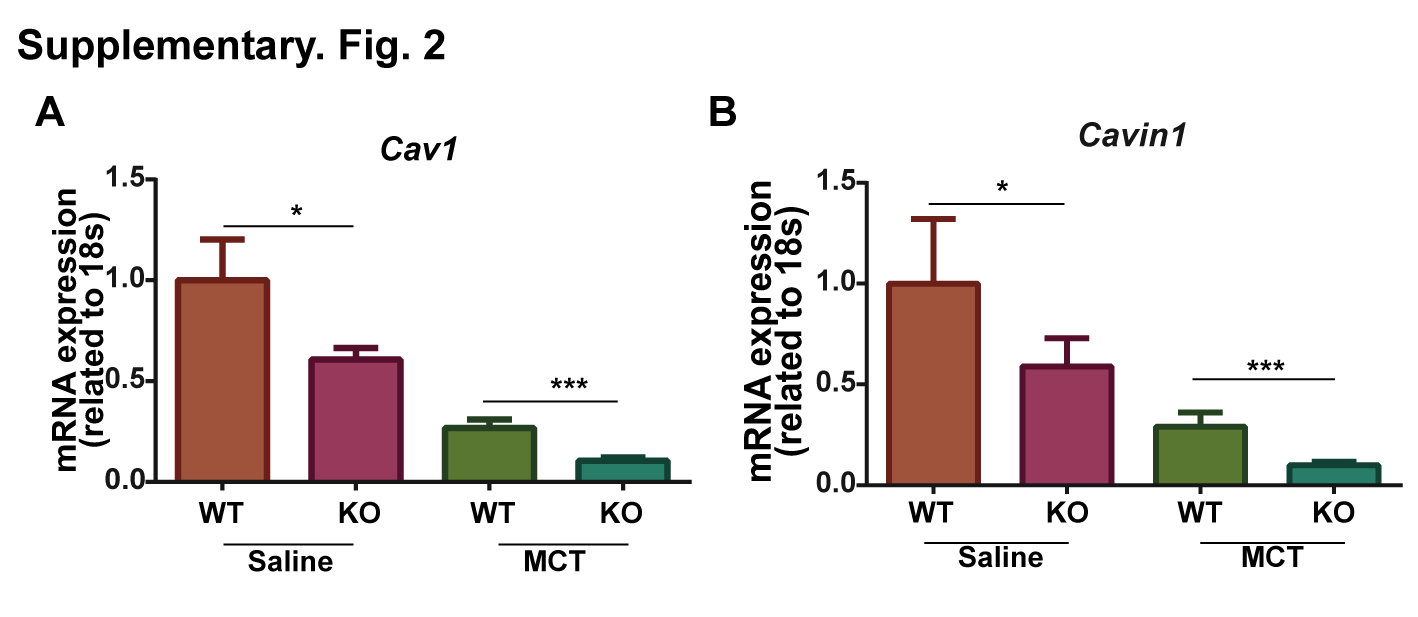

Supplement: Supplementary file 2 — Fig S2 [file JCMM-25-4732-s009.tif]

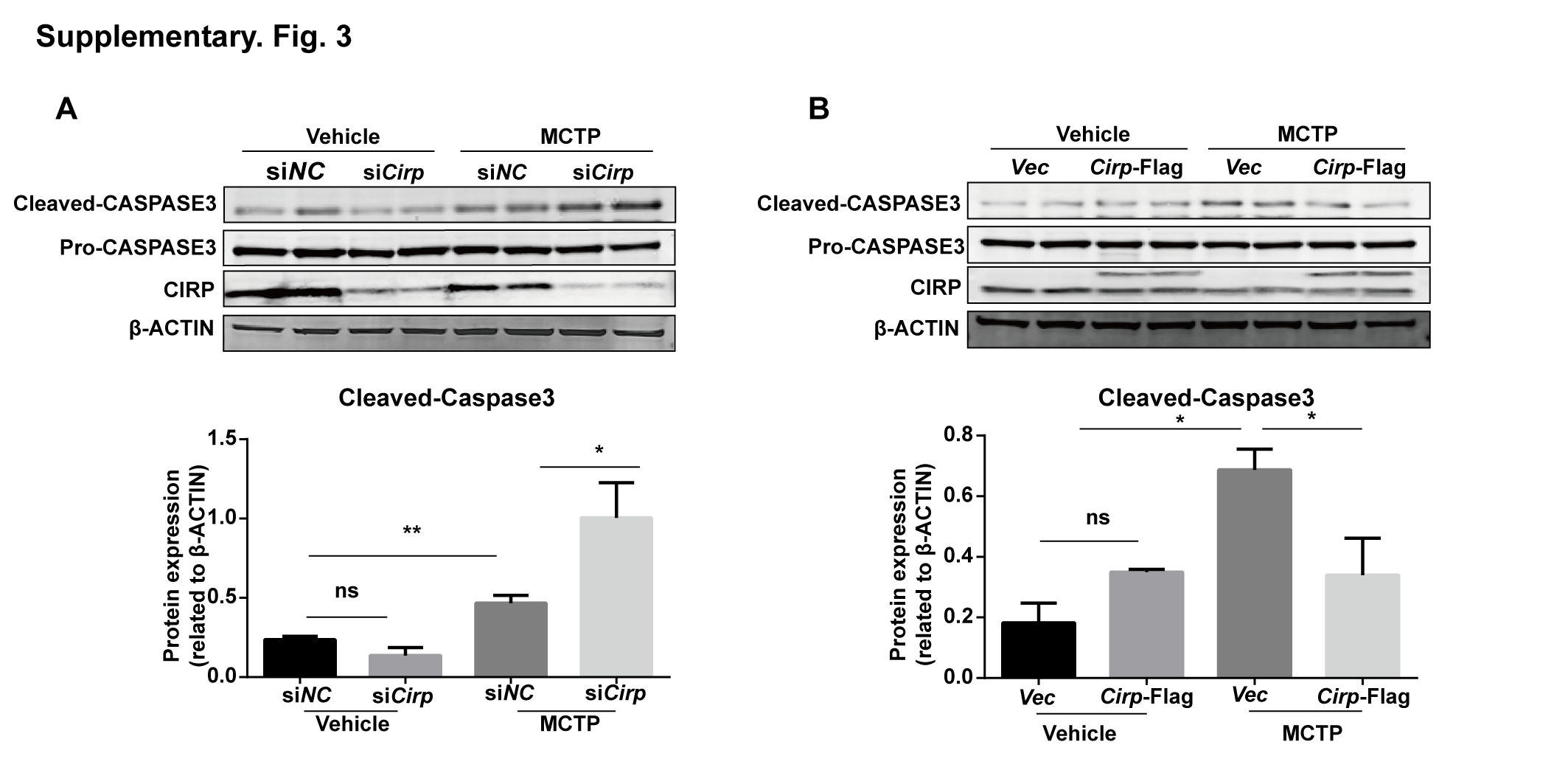

Supplement: Supplementary file 3 — Fig S3 [file JCMM-25-4732-s004.tif]

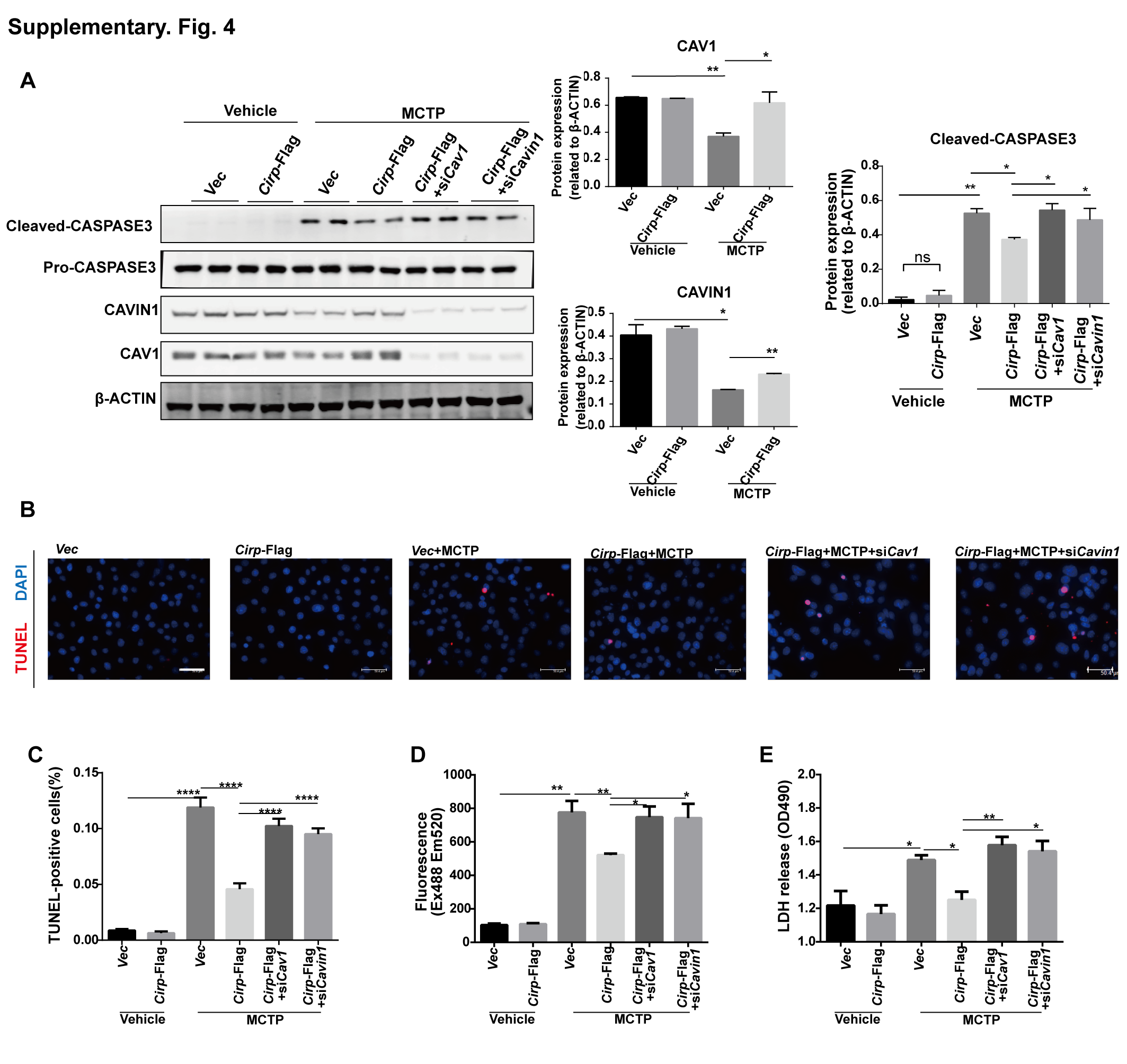

Supplement: Supplementary file 4 — Fig S4 [file JCMM-25-4732-s005.tif]

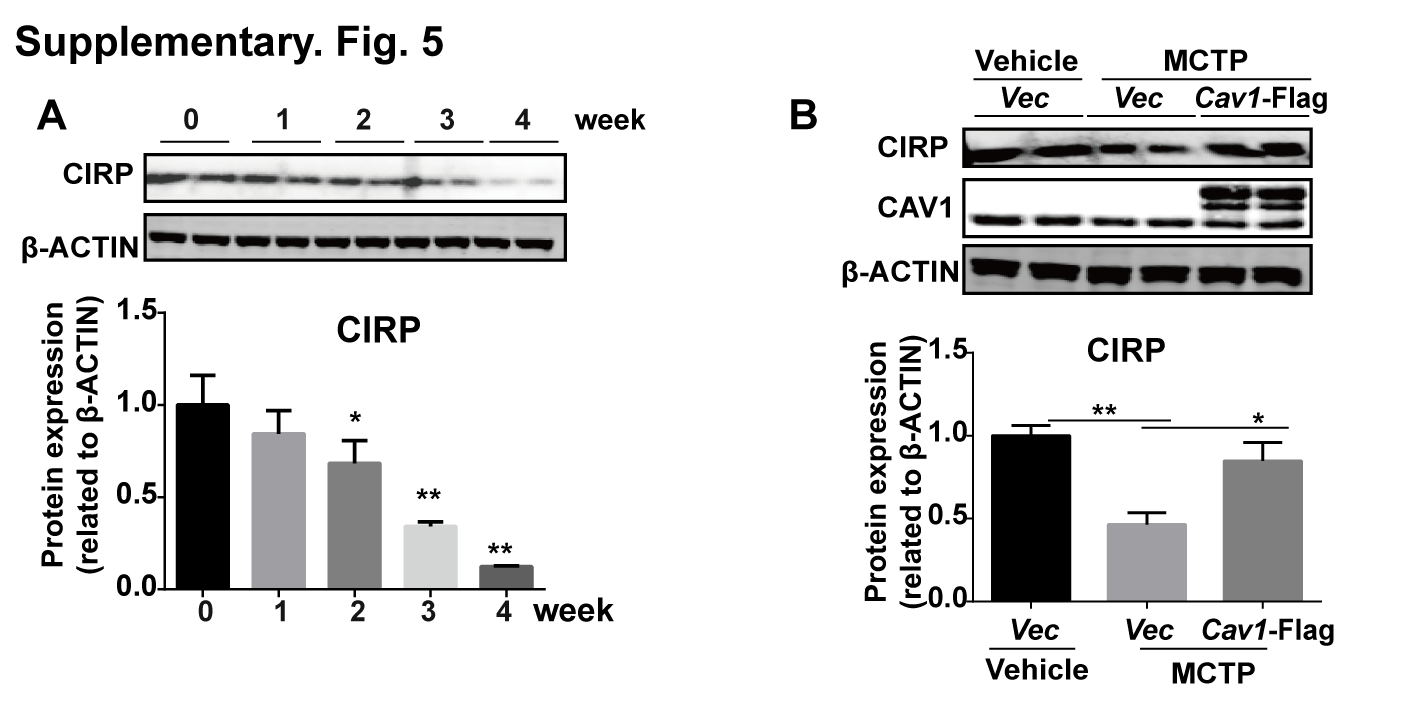

Supplement: Supplementary file 5 — Fig S5A‐B [file JCMM-25-4732-s007.tif]

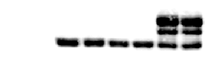

Supplement: Supplementary file 6 — Fig S5B‐wb‐CAV1‐adjusted [file JCMM-25-4732-s002.tif]

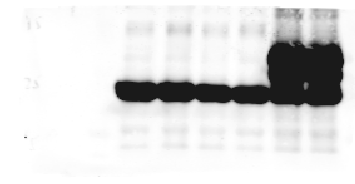

Supplement: Supplementary file 7 — Fig S5B‐wb‐CAV1 [file JCMM-25-4732-s001.tif]

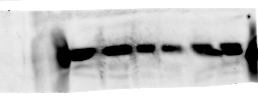

Supplement: Supplementary file 8 — Fig S5B‐wb‐CIRP [file JCMM-25-4732-s003.tif]

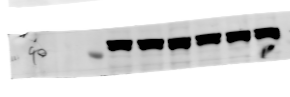

Supplement: Supplementary file 9 — Fig S5B‐wb‐OP¦‐ACTIN [file JCMM-25-4732-s008.tif]
